# Contribution of the Serotonin 5‐HT_2A_ Receptor to the Therapeutic Effect of Psilocin on Social Behavior Deficits in Mice Repeatedly Exposed to Social Defeat Stress

**DOI:** 10.1002/npr2.70152

**Published:** 2026-06-30

**Authors:** Daisuke Ibi, Rika Takaba, Keisuke Yoshida, Ririna Kawase, Hiroko Kitagawa, Momoko Matsushita, Kana Ito, Shoya Uno, Fumiya Nishimura, Shinji Kitagaki, Masayuki Hiramatsu

**Affiliations:** ^1^ Department of Chemical Pharmacology, Faculty of Pharmacy Meijo University Nagoya Japan; ^2^ Department of Chemical Pharmacology, Graduate School of Pharmacy Meijo University Nagoya Japan; ^3^ Department of Medicinal Chemistry, Faculty of Pharmacy Meijo University Nagoya Japan

**Keywords:** psilocin, serotonin 5‐HT_2A_ receptor, social defeat stress

## Abstract

Psychedelics such as psilocybin and lysergic acid diethylamide (LSD) exert hallucinogenic effects through stimulation of serotonin 5‐HT_2A_ receptors (5‐HT2ARs) in the cerebral cortex. In recent years, numerous reports have demonstrated that psychedelics are effective in treating various psychiatric disorders such as major depressive disorder (MDD), treatment‐resistant depression (TRD), and anxiety‐related disorders. We have previously reported that administration of psilocin, the active metabolite of psilocybin, produces antidepressant‐like effects in mice. Furthermore, we found that this effect is mediated by 5‐HT2AR activation. Since depression and other psychiatric disorders often lead to impairments in social behavior (e.g., social avoidance), the present study examined the effects of psilocin on social avoidance behavior in mice subjected to chronic social defeat stress (CSDS), a widely used model that closely models human psychosocial stress. Mice exposed to CSDS exhibited social avoidance behavior, whereas psilocin administration before the onset of CSDS had little effect on this behavior. In contrast, psilocin administration after the completion of CSDS ameliorated social avoidance in CSDS‐exposed mice. This effect was blocked by pretreatment with a 5‐HT2AR antagonist, indicating that psilocin exerts its therapeutic effects through 5‐HT2AR activation. Taken together, psilocin exerts therapeutic effects on social avoidance behavior after stress through activation of 5‐HT2AR, but not preventive effects when administered before stress, suggesting that psilocin may promote stress resilience rather than resistance.

## Abstract 2

Psilocin, the active metabolite of psilocybin, ameliorated social avoidance behavior in mice exposed to chronic social defeat stress when administered after, but not before, stress exposure via serotonin 5‐HT2A receptor activation. These findings suggest that psilocin exerts therapeutic, but not preventive, effects on stress‐induced social behavioral abnormalities.

## Graphical abstract

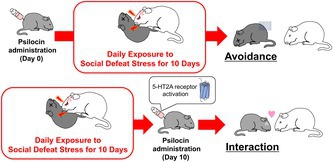

## 1 Introduction

Serotonergic psychedelics such as psilocybin—the hallucinogenic constituent of “magic mushrooms” with psilocin as its active metabolite—have been shown to induce rapid and sustained antidepressant effects in patients with major depressive disorder (MDD) and treatment‐resistant depression (TRD). The U.S. FDA has designated psilocybin/psilocin as a potential breakthrough therapy for MDD and TRD [1, 2]. We have previously reported that mice treated with psilocin show antidepressant‐like effects in forced‐swim and tail‐suspension tests, which depend on 5‐HT2AR activation [3]. Furthermore, the effects of psilocin persist for a prolonged period, despite its relatively short half‐life of only a few hours [4, 5].

Studies of social defeat in humans are typically conducted within the field of social psychology and often focus on bullying in schools or workplaces. Victims of bullying are known to experience depression, anxiety, social phobia (i.e., social avoidance), and other behavioral disturbances [6, 7]. In other words, chronic exposure to psychosocial stress is strongly implicated in the development of psychiatric disorders such as depression and anxiety disorders [8]. Conversely, the acquisition of stress resilience or resistance can help prevent and/or ameliorate such disorders and has been associated with antidepressant effects [9, 10, 11, 12]. These findings suggest that antidepressant treatment may influence stress susceptibility. In recent years, several rodent models of depression have been developed for screening antidepressants. Among these, animals exposed to chronic social defeat stress (CSDS) are considered one of the most models for studying depression and stress, due to their close resemblance to human psychosocial stress [13]; however, the preventive and/or ameliorative effect of psilocybin/psilocin on social avoidance behavior in CSDS animal models remains unknown.

In the present study, we examined whether psilocin has preventive or ameliorative effects on social avoidance behavior in mice subjected to CSDS; mice were administered psilocin either before the onset of CSDS or after its completion, and subsequently subjected to a social interaction test. Furthermore, the involvement of 5‐HT2AR in the effect of psilocin was also investigated. The present study aimed to clarify whether psilocin contributes to the acquisition of stress resistance and/or resilience.

## 2 Methods

### 2.1 Animals and Drugs

Male C57BL/6J mice (4–6 weeks old; Japan SLC, Shizuoka, Japan) and male ICR mice (8–20 weeks old; Japan SLC) were used in the present study. Animals were housed in a temperature‐controlled room (24°C ± 1°C) with a relative humidity of 55% ± 5% under a 12‐h light/dark cycle (lights on from 07:00 to 19:00). Psilocin and volinanserin, an antagonist of 5‐HT2AR, were prepared as previously described [3].

### 2.2 Establishment of the Social Defeat Stress

**FIGURE 1:**
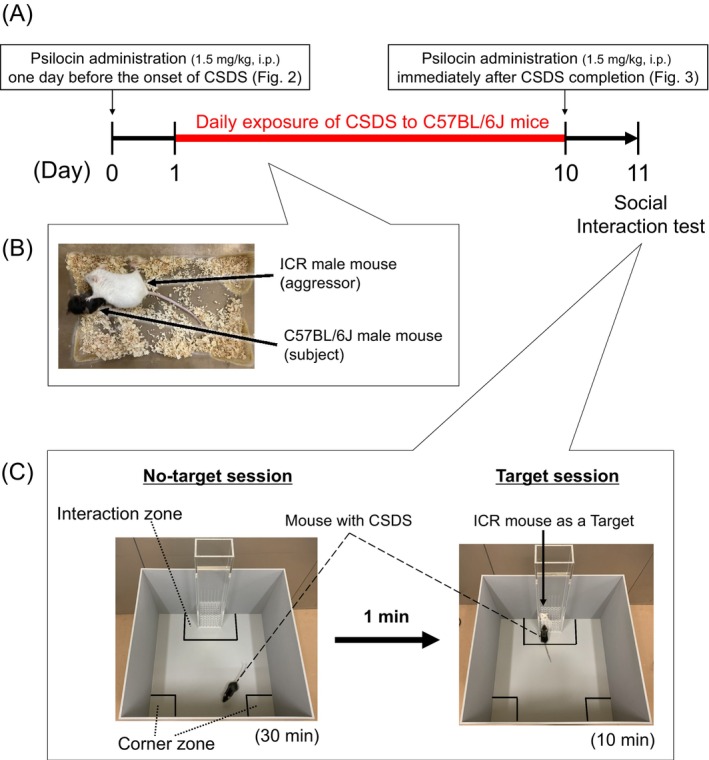
Experimental design illustrating the influence of social defeat stress exposure on social behavior. C57BL/6J mice were exposed to social defeat stress induced by an aggressive ICR mouse for 10 consecutive days (A, B). A social interaction test was conducted 1 day after the last stress exposure (A, C). Mice were intraperitoneally administered psilocin at 1.5 mg/kg 1 day before the onset of social defeat stress or immediately after the last stress exposure (A).

The CSDS model was established as previously described (see also Figure 1) [13]. Male ICR mice (8 weeks old) were individually housed in plastic cages (12.5 × 19.5 × 11 cm) for at least 7 days to enhance aggressive behavior. Male C57BL/6J mice (4–6 weeks old) were then exposed daily to an aggressive ICR mouse for up to 10 min, until the cumulative duration of aggressive encounters reached 1 min per day. This stress procedure was repeated for 10 consecutive days. To minimize variability in aggression exposure, the pairing of C57BL/6J and ICR mice was randomized each day.

### 2.3 Apparatus

The social interaction test was performed in a cubic open‐field arena (45 × 45 × 45 cm; Science Shokai, Nagoya, Japan). A transparent plastic cage was positioned against one wall of the arena. The area extending 5.8–6 cm from the cage was defined as the interaction zone, whereas two square areas (9 × 9 cm) in the corners opposite the interaction zone were defined as the corner zones (Figure 1C).

### 2.4 Procedure

On the day following the final SDS exposure, C57BL/6J mice were placed in the arena for 30 min in the absence of a target mouse in the transparent cage (No‐target session, Figure 1C). One minute later, a novel ICR mouse (not previously used in the SDS procedure) was placed inside the transparent cage, and the C57BL/6J mouse was allowed to explore freely in the arena for 10 min (Target session, Figure 1C). During both sessions, the time spent in the interaction and corner zones was recorded and analyzed using EthoVision XT (Noldus, Wageningen, the Netherlands). A social interaction (SI) ratio and corner zone ratio were then calculated according to the following:

The SI ratio and corner zone ratio were defined as the time spent in the interaction zone or corner zone during the 10‐min target session divided by the corresponding time during the last 10 min of the no‐target session, respectively.

### 2.5 Statistical Analysis

Statistical analyses and graph generation were performed using Prism 10 (GraphPad Software, San Diego, CA, USA). Behavioral data are presented as means ± SEM. Data were analyzed using one‐way ANOVA, followed by Bonferroni's post hoc test for multiple comparisons when appropriate. Adjusted *p* values for pairwise comparisons are shown in Table 1.

**TABLE 1:** Adjusted *p* values for multiple comparisons (Bonferroni post hoc test following one‐way ANOVA). Detailed statistical analyses for multiple comparisons are presented for all figures.

	Session or outcome measures	Adjusted *p* value		
		Control vs. CSDS	CSDS vs. CSDS+Psilocin	CSDS+Psilocin vs. CSDS+Psilocin+Volinanserin
Figure 2B	No‐target	> 0.9999	0.7296	
	Target	0.0001	0.2036	
Figure 2C	SI ratio	< 0.0001	0.1666	
Figure 2D	No‐target	> 0.9999	0.4736	
	Target	0.0002	0.0239	
Figure 2E	Corner zone ratio	0.0144	0.2642	
Figure 3B	No‐target	> 0.9999	> 0.9999	0.2757
	Target	< 0.0001	0.9353	0.0261
Figure 3C	SI ratio	< 0.0001	0.0082	0.0003
Figure 3D	No‐target	> 0.9999	0.7579	> 0.9999
	Target	< 0.0001	0.3253	< 0.0001
Figure 3E	Corner zone ratio	0.0156	0.1932	0.0902

## 3 Results

### 3.1 Effect of Psilocin Administration Before CSDS Onset on Social Avoidance Behavior in Mice With CSDS

**FIGURE 2:**
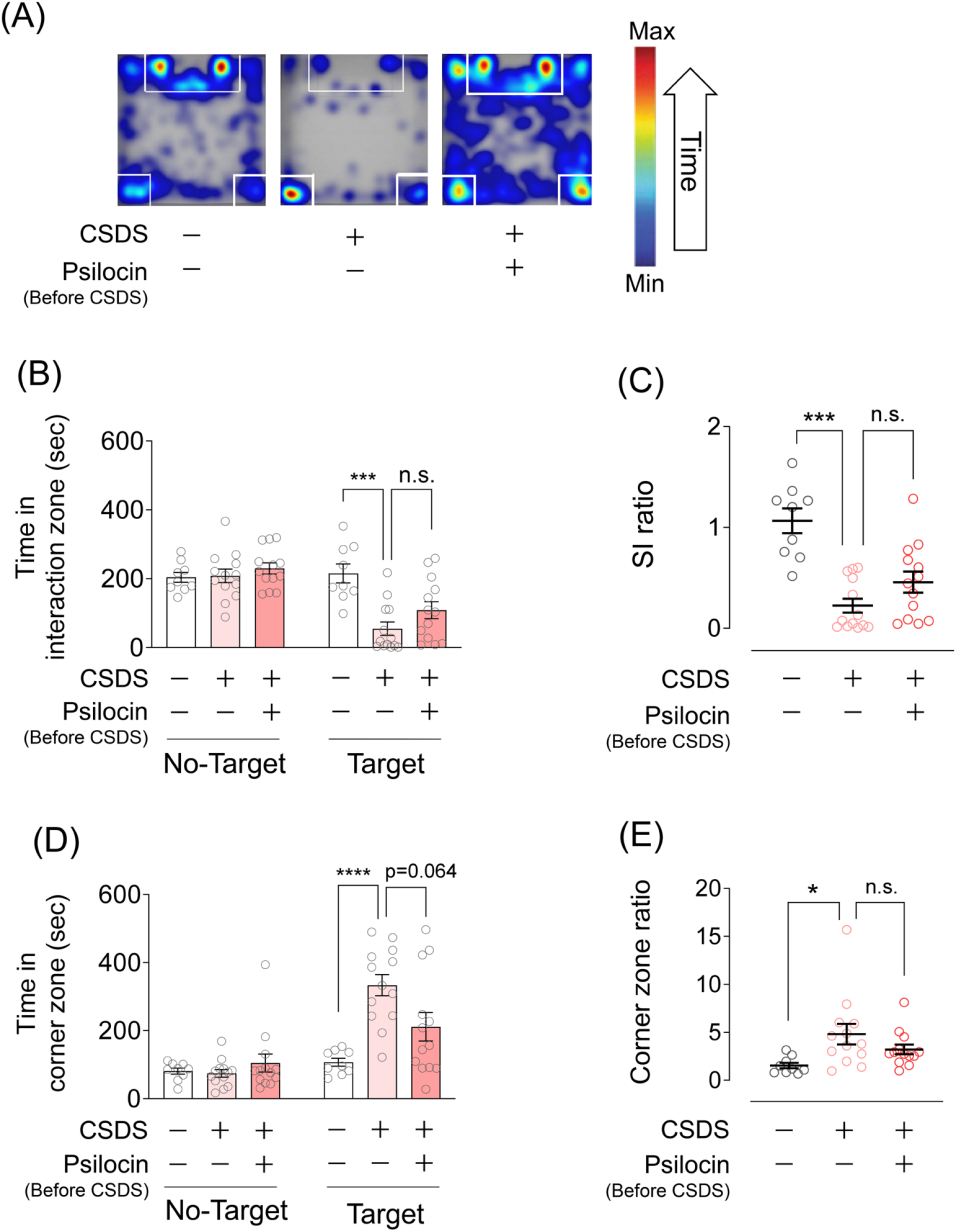
Effect of psilocin administration before the onset of CSDS exposure on the social avoidance in mice. C57BL/6J mice were exposed to aggressor ICR mice for 10 consecutive days. Vehicle or psilocin (1.5 mg/kg) was administered 1 day before the onset of CSDS (Before CSDS). The social interaction test was performed 1 day after the last CSDS session. Panels show representative heatmaps during the target session (A). The time spent in interaction (B) and corner (D) zones was measured by the social interaction test, and the SI (C) and corner zone (E) ratio were calculated. Values indicate the mean ± SEM (*n* = 9–13). Significance levels: *p < 0.05, ***p < 0.001, ****p < 0.0001 vs. control (Bonferroni's test). n.s., not significant.

To investigate the preventive effect of psilocin on social deficits in mice exposed to CSDS, psilocin was administered before the onset of CSDS exposure for 10 days, and social behaviors were subsequently examined (Figure 1A). Heatmaps were generated for each group during the target session (Figure 2A), where red indicates areas of prolonged occupancy and blue indicates shorter occupancy (Figure 2A). CSDS exposure decreased the time spent in the interaction zone during the target session, which was not influenced by psilocin administration before the onset of CSDS (Figure 2B, target session: *F*_(2,32)_ = 10.24, *p* = 0.0004; see Table S1), while there were no differences among three groups during the no‐target session (Figure 2B, no‐target session: *F*_(2,32)_ = 0.631, *p* = 0.539). Likewise, CSDS decreased the SI ratio; however, psilocin administration before the onset of CSDS had no effect (Figure 2C, *F*_(2,32)_ = 17.58, *p* < 0.0001; see Table S2).

On the other hand, CSDS increased the time spent in the corner zone during the target session, which tended to be suppressed by psilocin pretreatment (Figure 2D, target session: *F*_(2,32)_ = 10.24, *p* = 0.0004; see Table S3). As with the time in the interaction zone, neither CSDS nor psilocin administration affected the time in the corner zone during the no‐target session (Figure 2D, no‐target session: *F*_(2,32)_ = 0.786, *p* = 0.464). Furthermore, CSDS increased the corner zone ratio, but this was not statistically influenced by pretreatment with psilocin (Figure 2E: *F*_(2,32)_ = 4.156, *p* = 0.025; see Table S4).

These results suggest that CSDS impairs social behavior, which was not significantly affected by psilocin administration before the onset of CSDS.

### 3.2 Ameliorative Effect of Psilocin After CSDS Completion on Social Avoidance Behavior in Mice With CSDS

**FIGURE 3:**
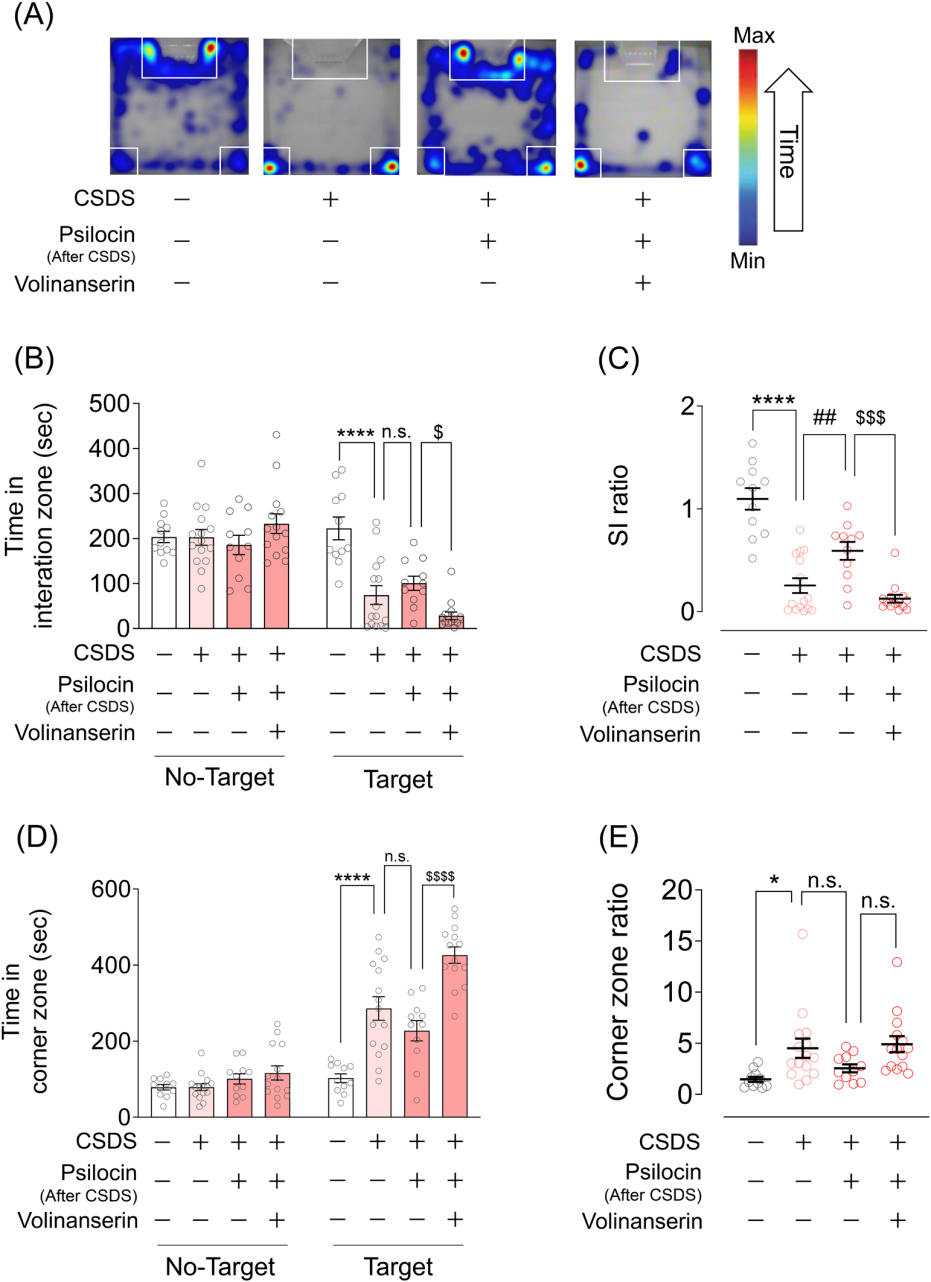
Effect of psilocin administration after completion of CSDS exposure on the social avoidance in mice. C57BL/6J mice were exposed to aggressor ICR mice for 10 consecutive days. Vehicle or psilocin (1.5 mg/kg) was administered immediately after CSDS completion (After CSDS). Volinanserin (Voli: 1.0 mg/kg) was administered intraperitoneally 30 min before psilocin administration. The social interaction test was conducted 1 day after the final CSDS session. Panels show representative heatmaps during the target session (A). The time spent in interaction (B) and corner (D) zones were measured by the social interaction test and the SI (C) and corner zone (E) ratio were calculated. Values indicate the mean ± SEM (*n* = 11–15). Significance levels: *p < 0.05, ****p < 0.0001 vs. control; ^##^ *p* < 0.01 vs. CSDS + vehicle; ^$^ *p* < 0.05, ^$$$^ *p* < 0.001, ^$$$$^ *p* < 0.0001 vs. CSDS + psilocin (After CSDS) (Bonferroni's test). n.s., not significant.

Next, to examine the ameliorative effect of psilocin on CSDS‐induced social avoidance behavior, mice were treated with psilocin immediately after completion of the CSDS procedure and were then subjected to the social interaction test (Figure 1A). Heatmaps illustrating the location of mice during the target session were generated, with red indicating areas of prolonged occupancy and blue indicating shorter occupancy (Figure 3A).

The time spent in the interaction and corner zones was measured during the no‐target and target sessions (Figure 3B,D). CSDS decreased the time spent in the interaction zone during the target session, which agrees with Figure 2. This was slightly, but not significantly, improved by psilocin administration immediately after the completion of CSDS; however, this psilocin effect was suppressed by volinanserin treatment (Figure 3B, target session: *F*_(3,47)_ = 19.09, *p* < 0.0001; see Table S5). During the no‐target session, neither CSDS nor psilocin administration affected the time spent in the interaction zone (Figure 3B, no‐target session: *F*_(3,47)_ = 1.065, *p* = 0.373). As shown in Figure 2, the SI ratio was also calculated in Figure 3C. The CSDS‐induced decrease in the SI ratio was improved by psilocin administration; however, this psilocin effect was absent by volinanserin administration (Figure 3C: *F*_(3,47)_ = 31.66, *p* < 0.0001; see Table S6).

Time spent in the corner zone was also measured, which was increased by CSDS during the target session; however, psilocin had no effect on time spent in the corner zone (Figure 3D, target session: *F*_(3,47)_ = 27.83, *p* < 0.0001; see Table S7). There were no differences among all groups in time spent in the corner zone during the no‐target session (Figure 3D, no‐target session: *F*_(3,47)_ = 2.007, *p* = 0.126). Regarding the ratio of time spent, CSDS increased the corner zone ratio, which was not statistically influenced by either psilocin or volinanserin (Figure 3E: *F*_(3,47)_ = 4.790, *p* = 0.0054; see Table S8).

These results suggest that psilocin treatment ameliorates social avoidance behaviors caused by CSDS, dependent on 5‐HT2AR activation.

## 4 Discussion

In rodent experiments involving social defeat stress, mice chronically exposed to this stress are generally classified as either susceptible or resilient [13]. For this classification, the social interaction test following social defeat stress is the most widely used behavioral paradigm, as employed in the present study [14]. Specific individuals are more vulnerable to stress and are thus classified as stress‐susceptible, whereas others display stress resilience and are less affected by stress exposure. In general, approximately half of the mice exposed to social defeat stress exhibit reduced social behavior and are classified as susceptible, while the remaining half do not show these changes and are considered resilient [15]. On the other hand, given the experimental schedule in which psilocin was administered before CSDS onset or immediately after its completion, the present study could not distinguish mice exposed to CSDS into susceptible and resilient groups. Furthermore, several previous studies have identified mice with an SI ratio of less than one as susceptible, and those with a ratio greater than one as resilient [13]. Interestingly, as shown in Figures 2C and 3C, all mice exposed to CSDS exhibited an SI ratio of less than one, suggesting that these mice may have been susceptible under the present experimental conditions. This finding is inconsistent with previous studies that categorized animals as either susceptible or resilient, and this discrepancy may be attributed to differences in animal age or in the intensity and duration of the defeat stress, as has been similarly reported in previous research [16].

Figure 2 shows that social avoidance behavior was little affected by a single dose of psilocin administration prior to CSDS onset. Considering that the antidepressant effects of a single dose of psilocin/psilocybin have been reported to persist for several weeks in rodents [3, 17], a single administration of psilocin before CSDS onset may be sufficient to affect social behavior after the completion of CSDS, although psilocin is known to be rapidly cleared from circulation [4, 5]. On the other hand, it may also be worthwhile to investigate whether daily administration of psilocin before each social defeat stress session over the 10‐day period affects social interaction behavior.

Conversely, CSDS‐induced social avoidance behavior was significantly ameliorated by psilocin administration immediately after the completion of CSDS; however, this psilocin effect was suppressed by volinanserin treatment. Taken together, psilocin might promote stress resilience through 5‐HT2AR activation, without affecting stress resistance. Additionally, the present study demonstrated that the therapeutic effect of psilocin is dependent on 5‐HT2AR; however, the hallucinogenic effects of psychedelics are also attributed to 5‐HT2AR [3, 18]. The molecular mechanisms underlying the therapeutic and hallucinogenic effect of psilocin via 5‐HT2AR remain unclear. Therefore, future research employing diverse behavioral and biochemical approaches will be required to elucidate the underlying mechanisms of psilocin and to further determine whether other psychedelics exert such effects through similar or distinct pathways.

It seems that volinanserin treatment may exacerbate CSDS‐induced social deficits beyond those observed in the CSDS only group in Figure 3B,D. However, we have previously reported that volinanserin itself has no effect on depressive‐ or anxiety‐like behaviors in mice not exposed to stress, suggesting that 5‐HT2AR antagonism itself unlikely affects emotional behaviors [3]. On the other hand, previous studies have shown that chronic stress, including social defeat stress, increases the expression of 5‐HT2AR in the prefrontal cortex (PFC), a brain region involved in antidepressant effects [19], suggesting that chronic stress leads to alterations in 5‐HT2AR signaling. These raise the possibility that 5‐HT2A antagonism itself may influence behavioral outcomes in mice under chronic stress conditions. Accordingly, further studies are required to investigate the effect of volinanserin alone on social avoidance behavior in mice exposed to CSDS.

## Author Contributions

Conceptualization: Daisuke Ibi. Experiments and data analysis: Daisuke Ibi, Rika Takaba, Ririna Kawase, Hiroko Kitagawa, Momoko Matsushita, Kana Ito, Shoya Uno, and Fumiya Nishimura. Synthesis of psilocin: Keisuke Yoshida and Shinji Kitagaki. Writing: Daisuke Ibi, and Masayuki Hiramatsu. Supervision, Daisuke Ibi, and Masayuki Hiramatsu. All authors have read and approved the final manuscript.

## Funding

This work was supported by research grants from the SRF, NOVARTIS Foundation (Japan) for the Promotion of Science, the Hori Science and Arts Foundation, the Takahashi Industrial Economic Research Foundation, and JSPS KAKENHI grants 22K06872 and 26K09675.

## Conflicts of Interest

The authors declare no conflicts of interest.

## Supporting information


**Table S1:** Time spent in the interaction zone in mice administered psilocin before the onset of CSDS exposure. The raw data for Figure 2B are presented in this table.
**Table S2:** SI ration in mice administered psilocin before the onset of CSDS exposure. The raw data for Figure 2C are presented in this table.
**Table S3:** Time spent in corner zone in mice administered psilocin before the onset of CSDS exposure. The raw data for Figure 2D are presented in this table.
**Table S4:** Corner zone ratio in mice administered psilocin before the onset of CSDS exposure. The raw data for Figure 2E are presented in this table.
**Table S5:** Time spent in the interaction zone in mice administered psilocin after CSDS exposure (Figure 3B). The raw data for Figure 3B are presented in this table.
**Table S6:** SI ratio in mice administered psilocin after CSDS exposure. The raw data for Figure 3C are presented in this table.
**Table S7:** Time spent in corner zone in mice administered psilocin after CSDS exposure. The raw data for Figure 3D are presented in this table.
**Table S8:** Corner zone ratio in mice administered psilocin after CSDS exposure. The raw data for Figure 3E are presented in this table.

## Data Availability

The raw data of this study are presented as (Tables S1–S8).
